# Asthma: A Gut Reaction to Antibiotics

**Published:** 2005-06

**Authors:** Carol Potera

Is the explosive rise in asthma and allergies being seen especially in children partially related to antibiotic use? Epidemiologic studies have found strong connections between antibiotic treatment and the later development of asthma and allergies. Yet, until recently, no studies had looked at how the two are linked. Now researchers at the University of Michigan in Ann Arbor have created a mouse model that offers clues to the mechanism behind the association.

Immunologist Gary Huffnagle and colleagues are the first to demonstrate in a mouse model that the disruption of beneficial intestinal bacteria by antibiotics allows yeast to take hold and flourish. They developed their mouse model specifically to study the relationship between antibiotic use and allergies. When mice inhale fungal spores known to trigger allergies in people, the allergic reaction is more potent in mice with an overgrowth of yeast in their guts.

In their studies, the Michigan researchers first treat mice for several days with the broad-spectrum antibiotic cefoperazone to destroy the gut flora. Then the mice are fed *Candida albicans*, a yeast that commonly lives in people. “This represents the clinical scenario of getting a yeast infection after taking antibiotics,” says Huffnagle. Next, the mice are exposed nasally to spores of the mold *Aspergillus fumigatus* (a major indoor contaminant) and to egg white protein.

Results are showing that both allergens produce significant increases in inflammation-related white blood cells in the lungs of the mice, and they elevate blood levels of key markers of allergic reactions, including IgE, interleukin-5, and interleukin-13. Mice not treated with antibiotics show much milder reactions to the allergens. The team’s latest report appears in the January 2005 issue of *Infection and Immunity*. Future work with the model will investigate the actions of other antibiotics (such as amoxicillin) and allergens (such as pollen and dust mites).

How do changes in gut flora influence respiratory allergies? The answer likely involves oral tolerance, Huffnagle theorizes. Upon ingestion of allergens, the oral mucosa generate regulatory T cells, which circulate to the respiratory tract, where they suppress allergic reactions. “We live in a dirty world, and we swallow mold spores, pollen, dust, and other allergens constantly,” says Huffnagle. These oral allergens trigger immune responses that instruct the rest of the body to be more tolerant of allergens so allergic reactions don’t occur. Moreover, other studies have indicated that mice lacking gut flora cannot generate oral tolerance. When the gut flora are restored, oral tolerance returns.

Huffnagle plans to evaluate over-the-counter probiotics—concentrated supplements of beneficial bacteria—to identify which, if any, work best for replenishing gut flora. “[Probiotics are a] relatively new concept, and there’s not a lot of precedent for their use now,” says infectious disease specialist Bruce Klein of the University of Wisconsin–Madison. If future studies show that probiotics do replace flora, Klein adds, physicians may be inclined to recommend their use. Eating yogurt with live cultures also remains a good way to replenish gut flora following a course of antibiotics.

